# Analysis and validation of novel biomarkers related to palmitoylation in adenomyosis

**DOI:** 10.3389/fgene.2025.1614573

**Published:** 2025-08-04

**Authors:** Hongyu Zhang, Yufeng Li, Huijuan Cao, Yiling Zhao, Hongwen Zhu, Tiansheng Qin

**Affiliations:** ^1^Department of Gynecology, The Second Hospital & Clinical Medical School, Lanzhou University, Lanzhou, China; ^2^Cuiying Biomedical Research Center, The Second Hospital & Clinical Medical School, Lanzhou University, Lanzhou, China

**Keywords:** palmitoylation, adenomyosis, machine learning, diagnostic model, gene expression

## Abstract

**Background:**

Adenomyosis, a common gynecological disorder in women of reproductive age, is characterized by endometrial invasion into the myometrium, leading to uterine enlargement and smooth muscle hypertrophy. Typical clinical symptoms include chronic pelvic pain, abnormal uterine bleeding, and infertility, which significantly impair patients’ quality of life. Currently, effective diagnostic biomarkers for adenomyosis are lacking. Recent studies suggest that estrogen may promote Scribble protein depalmitoylation by upregulating APT1 and APT2 expression. Depalmitoylation facilitates Scribble’s translocation from the cell membrane to the cytoplasm, disrupting endometrial epithelial cell polarity. This polarity loss may enhance abnormal proliferation, migration, and invasion of endometrial epithelial cells, promoting endometrial tissue infiltration into the myometrium and contributing to adenomyosis development and progression. Therefore, investigating adenomyosis diagnosis and treatment from the perspective of palmitoylation-related genes holds significant scientific importance.

**Methods:**

In this study, four datasets, GSE244236, GSE190580, GSE185392 and GSE157718, were downloaded and the data were screened and standardized the data. First, GSE244236 was used as the training dataset. By integrating multiple bioinformatics approaches—including differential gene analysis (DEGs), weighted gene co-expression network analysis (WGCNA), Least Absolute Shrinkage (LASSO), random forest (RF) methods, and Support Vector Machine-recursive feature elimination (SVM-RFE)—we identified three overlapping diagnostic genes through comprehensive analysis. Meanwhile, the diagnostic value of each biomarker was assessed using the receiver operating characteristic curve analysis in the remaining three datasets. In addition, single-sample gene set enrichment analysis (ssGSEA) were utilized to explore the infiltration of immune cells in adenomyosis and to examine the correlation between diagnostic biomarkers and immune cells.

**Results:**

A total of 549 differentially expressed genes were identified in the analysis. Through WGCNA analysis, we obtained 25 palmitoylation-related intersecting genes. Using LASSO, RF and SVM-RFE algorithms, three potential diagnostic genes were finally screened: LIPH, CYP2E1 and CHRNE.

**Conclusion:**

In this study, we successfully identified diagnostic biomarkers for adenomyosis using comprehensive bioinformatics analysis and machine learning methods, and validated them with nomogram and ROC curves. Our findings provide new perspectives for understanding the pathogenesis of palmitoylation-related genes in adenomyosis and offer potential targets for the development of new therapeutic strategies.

## 1 Introduction

Adenomyosis is a common benign condition in women of childbearing age that is characterized by the invasion of the endometrium into the myometrium, causing hyperplasia, fibrosis and uterine enlargement. Although the exact etiology and pathogenesis of adenomyosis are not fully understood, it is widely believed to be related to an estrogen imbalance, immune-inflammatory response, angiogenesis, and imbalance in cell proliferation and apoptosis. Traditional diagnostic methods include ultrasonography, biomarkers, and clinical symptom evaluation. With continuous advances in imaging technology, transvaginal ultrasonography (TVUS) and magnetic resonance imaging (MRI) have been shown to be valuable in the diagnosis of adenomyosis (Dason et al., 2023; Krentel et al., 2023). However, the treatment of adenomyosis has received increasing attention due to the lack of effective diagnostic markers and the side effects associated with drug therapy. In recent years, with the limitations of traditional treatments, the development of new technologies, and innovations in bioengineering techniques, novel diagnostic and therapeutic methods have emerged, including precision imaging techniques, infrared fluorescence imaging, magnetic resonance imaging, and photoacoustic imaging, which have enhanced the visualization of lesions and improved surgical precision. In addition, new methods such as bioengineered drug delivery systems, immunotherapy, gene therapy, ferroptosis induction and synthetic lethal activation provide new avenues for the effective treatment of adenomyosis (Peng et al., 2025). These methods offer certain advantages in precise diagnosis, treatment, and maintenance of fertility in women of childbearing age, which can help to advance precision medicine and improve women’s health. Therefore, it is particularly important to identify the pathogenesis of adenomyosis and explore potential therapeutic targets, which can help to improve the success rate of treatment and reduce side effects.

Palmitoylation is a reversible post-translational modification that adds palmitic acid, a 16-carbon palmitic acid, to a cysteine residue via a thioester bond. Palmitoylation is catalyzed by a family of zinc finger-containing DHHC-type proteins (ZDHC1-9, ZDHC11-24), whereas depalmitoylation is catalyzed by acyl-protein thioesterases (APT1/2), palmitoyl-protein thioesterases (PPT1/2), or by proteins containing the structural domains of the α/β-hydrolase domain-containing proteins 17A/B/C (ABHD17A/B/C) (Ko and Dixon, 2018; Zhou et al., 2023). By modulating protein localization, signaling pathways, and protein-protein interactions, palmitoylation influences cancer progression, neurodegeneration, inflammation, and metabolic disorders (Chen et al., 2017; Kong Y. et al., 2023; Zhang et al., 2025). It also regulates immune cell activation and inflammatory factor secretion, making palmitoylation enzymes potential therapeutic targets (Blaskovic et al., 2013; De and Sadhukhan, 2018; Kong Y. et al., 2023; Main and Fuller, 2022; Won et al., 2018; Zeng et al., 2024). Notably, in adenomyosis, estrogen may upregulate APT1/2, promoting Scribble depalmitoylation and its translocation from the membrane to the cytoplasm, thereby disrupting epithelial polarity and contributing to disease pathogenesis (Jin et al., 2024). Similarly, in endometriosis, ZDHHC12-mediated palmitoylation of NLRP3 facilitates its autophagic degradation, which modulates inflammatory responses. Furthermore, analysis shows that palmitoylation-related genes are correlated with immune cell infiltration, including M2 macrophages and resting NK cells (Kai et al., 2025). These findings suggest that palmitoylation plays a multifaceted role in the molecular mechanisms underlying adenomyosis and related diseases, providing potential targets for therapeutic intervention.

Recent studies have identified several potential biomarkers for adenomyosis. Elevated serum CA125 levels correlate with disease severity, while mild increases in CA199 and CEA may reflect inflammatory responses or lesion extent (Chen W. C. et al., 2022; Liu et al., 2025). Urinary miR-92a-3p promotes tumor infiltration and angiogenesis, and serum creatinine phosphokinase has been proposed as a noninvasive diagnostic marker (Shao et al., 2025). The serum creatine phosphokinase level has been suggested as a noninvasive diagnostic marker (Bulut Aydemir et al., 2025). Additionally, exosomal HSP90A, STIP1, and TAGLN-2 are specifically upregulated in adenomyosis, suggesting disease-specific pathological processes (Chen D. et al., 2022). Transcriptomic and genomic analyses further reveal KRAS mutations and increased RhoA-ROCK signaling, which enhance cell survival, proliferation, and progesterone resistance (Bulun et al., 2021; Jiang et al., 2018; Xiang et al., 2019). Single-cell sequencing has also uncovered significant fibroblast heterogeneity in adenomyotic uteri (Yildiz et al., 2023). Collectively, these findings highlight the diagnostic potential of biomarkers and underscore the importance of genomic studies in elucidating adenomyosis pathogenesis.

This study applied comprehensive bioinformatics analysis and machine learning algorithms to identify diagnostic biomarkers and explore immune infiltration in adenomyosis. Four datasets of adenomyosis were downloaded from the Gene Expression Omnibus (GEO) database as training and validation sets. Diagnostic biomarkers were identified by differential gene analysis, integration of WGCNA, and palmitoylation-related gene sets to take the intersection, followed by the joint LASSO, RF and SVM-RFE algorithms. ssGSEA was used to identify differences in the infiltration of endometrial immune cells and the correlation between diagnostic biomarkers and immune cells in women with adenomyosis and controls.

## 2 Materials and methods

### 2.1 Data set collection and processing

We obtained the gene expression profiles of adenomyosis from the GEO database, including four microarray datasets: GSE244236, GSE190580, GSE185392, and GSE157718. The GSE244236 dataset contains the expression profiles of 28 patients with adenomyosis and 25 normal controls. The remaining three datasets contain endometrial expression profiles of different numbers of adenomyosis patients and controls, respectively. We set GSE244236 as the training set and the remaining three datasets as the validation set. In addition, we downloaded 3228 palmitoylation-related genes through the GeneCards website (Zeng et al., 2024).

### 2.2 Identification and functional enrichment analysis of differentially expressed genes

After performing gene re-annotation on the dataset probes using platform files, all data underwent logarithmic transformation and normalization through the normalizeBetweenArrays function. The Limma package was then employed to identify DEGs between 28 women with adenomyosis and 25 healthy controls, using the threshold for significant differences at adjusted p-values <0.05 and |log2(FC)| ≥ 1, which resulted in the identification of 549 DEGs (Figure 2A). To provide a visual overview of these DEGs, subsequent visualization involved volcano plot analysis (bioinformatics.com.cn). In order to understand the functional implications of these DEGs, we conducted functional enrichment analyses. Through Gene Ontology (GO) analysis, we examined biological processes (BP), cellular components (CC), and molecular functions (MF) associated with the DEGs. Additionally, Kyoto Encyclopedia of Genes and Genomes (KEGG) enrichment analysis was applied to explore pathway enrichment (Figures 2B–D).

### 2.3 Weighted gene co-expression network analysis

WGCNA represents a systematic biological approach for constructing gene co-expression networks, clustering genes with similar expression patterns, and exploring network modules closely related to clinical features. Therefore, we utilized the oebiotech platform (https://www.oebiotech.com) to build a gene co-expression matrix from the GSE244236 dataset. Following the principle of scale-free networks, we selected a soft threshold (power = 24, *R* ^2^ = 0.81) to sequentially construct a scale-free co-expression network, converting the adjacency matrix into a topological overlap matrix. Subsequently, cluster analysis was performed to identify gene modules, with each module comprising at least 50 genes. Hierarchical clustering was used to construct dendrograms and to calculate correlations between characteristic genes in the modules and disease phenotypes. By integrating multiple metrics such as correlation coefficients, p-values, and the GS and MM values of the modules, we ultimately identified the module most positively correlated with adenomyosis as a key disease-associated module.

### 2.4 Screening candidate diagnostic biomarkers using machine learning algorithms

After taking intersections with the MElightyellow modular genes identified by WGCNA, alongside 3,228 palmitoylated genes, and 549 DEGs (Figure 4A), we identified 25 palmitoylated genes that were differentially expressed in adenomyosis. To refine the identification of characteristic genes, we employed three machine learning algorithms: Random Forest (RF), Support Vector Machine (SVM) with Recursive Feature Elimination (RFE), and LASSO logistic regression. LASSO effectively filters out redundant features by applying an L1 penalty (λ) to compress insignificant variable coefficients to zero, thereby refining the model. A higher λ value reduces the number of selected variables, making key genes more representative of the disease state (Wang et al., 2023). SVM-RFE, a supervised learning technique, identifies core candidate genes by gradually eliminating features with minimal contribution to model performance. RF evaluates variable importance using decision trees, facilitating feature prioritization (Kong X. et al., 2023; Xu et al., 2023). The final candidate diagnostic biomarkers were determined by intersecting the results of these three methods, ensuring a comprehensive and reliable screening process. These complementary algorithms collectively enhance both the accuracy and robustness of gene feature selection.

### 2.5 Validation of ROC curves for diagnostic markers

To evaluate the diagnostic efficacy of the candidate biomarkers, we analyzed and compared the expression levels of the three key genes in the adenomyosis group versus the control group using the oebiotech Platform, with box plot visualization. Meanwhile, the CNSknowall platform (https://cnsknowall.com) was employed to conduct ROC curve analysis and joint assessments for each hub gene, calculating the area under the curve (AUC) with a 95% confidence interval (CI). Significance was determined based on AUC values, where values closer to 1 indicate greater accuracy in distinguishing between groups during model evaluation.

### 2.6 Immune cell infiltration analysis

To investigate differences in the functional activity of immune cells between adenomyosis patients and healthy controls, we conducted single-sample gene set enrichment analysis comparing the adenomyosis group and control group. Figure 8 presents heat maps illustrating differential expression levels of 28 immune cell types within the GSE244236 dataset comparing adenomyosis patients and controls, while box plots demonstrate the distribution patterns of 16 specific immune cell infiltrations and 13 immune-related biological markers.

### 2.7 Correlation analysis between diagnostic biomarkers and infiltrating immune cells

The correlations between the three diagnostic biomarkers initially identified and the infiltrating immune cells were evaluated using a heatmap, which provides a clear visualization of their respective relationships with immune cell infiltration.

## 3 Results

### 3.1 Screening and visualization of differentially expressed genes

**FIGURE 1:**
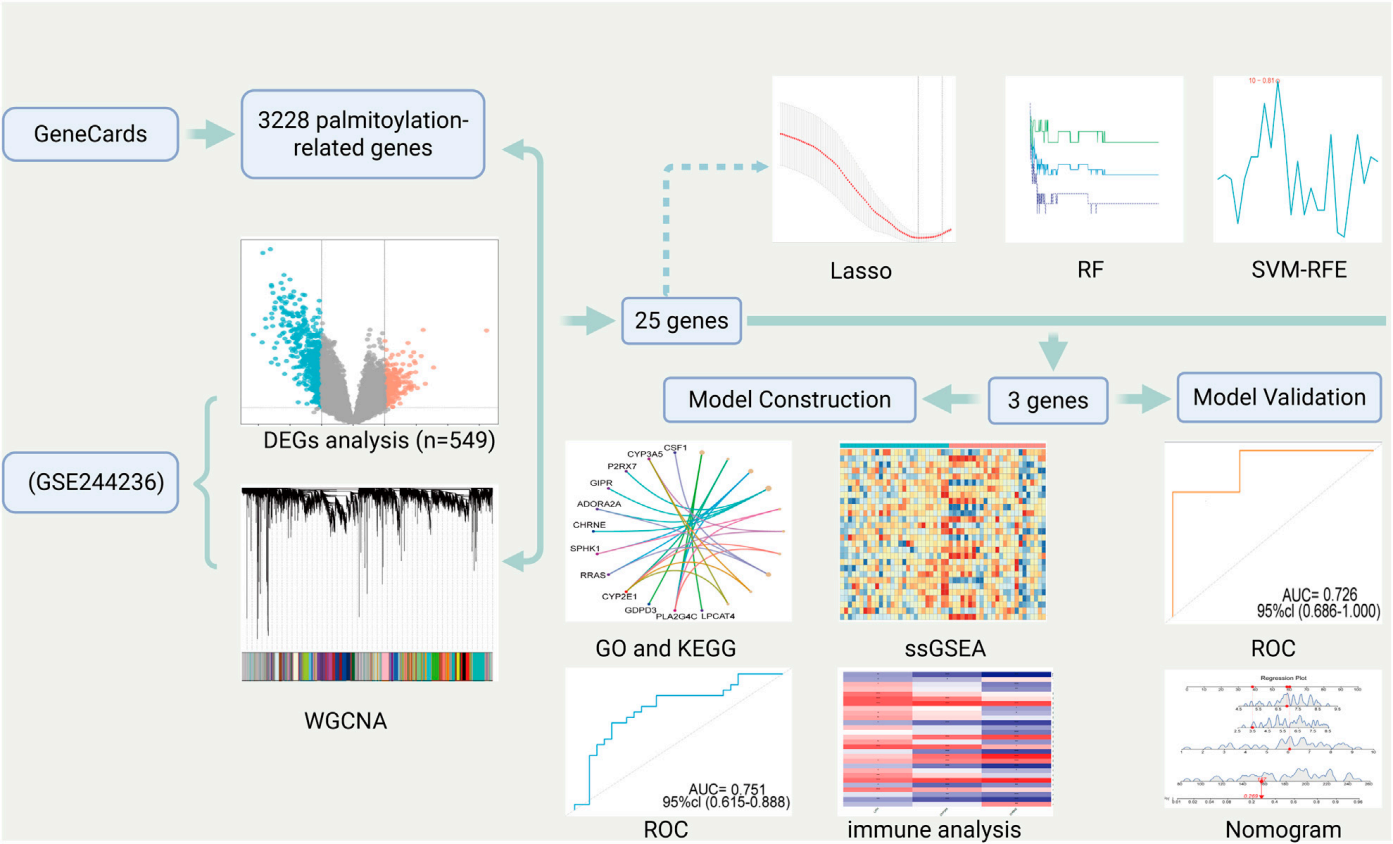
Provides a flowchart of the entire workflow and study design. (Created by BioRender).

The flow of this study is shown in Figure 1.

**FIGURE 2:**
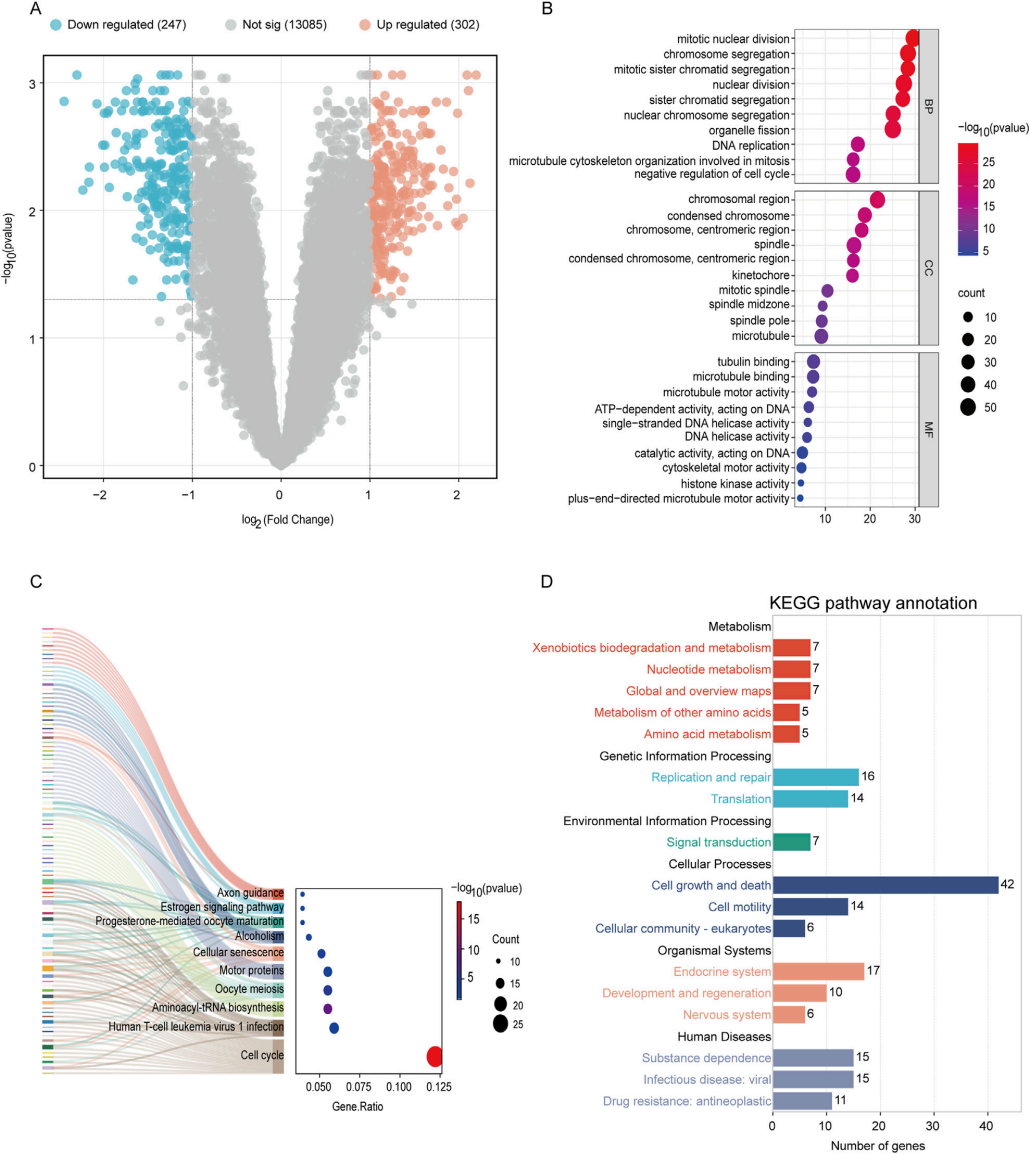
Analysis of DEGs profile in endometrium between women with adenomyosis and controls. **(A)** Volcano plot showing DEGs in adenomyosis patients versus healthy control. The red dots represent the upregulated genes and the green dots represent the downregulated genes in the adenomyosis group. (|log2 FC |≥1; adjusted p-value <0.05). **(B)** GO enrichment analysis of DEGs. The top 10 BP, MF, and CC terms of DEGs. **(C–D)** The KEGG pathway enrichment analysis of DEGs.

The dataset GSE244236 was sourced from the Gene Expression Omnibus (GEO) database. Using probe annotation information, we converted integrated IDs in the gene expression matrix into gene symbols. The expression matrices were then normalized through log2 transformation and processed using the normalizeBetweenArrays function. Differential analysis was performed with the limma package to compare 28 adenomyosis patients with 25 normal controls, identifying 549 DEGs with adjusted p-values <0.05 and |log2(FC)| ≥ 1, visualized through volcano plots (Figure 2A). Among these DEGs, 302 genes showed upregulation (indicated by red dots), while 247 showed downregulation (indicated by blue dots). To elucidate their functional significance, we conducted Gene Ontology (GO) and Kyoto Encyclopedia of Genes and Genomes (KEGG) pathway enrichment analyses using platform (http://www.bioinformatics.com.cn), with a significance threshold of P < 0.05.

Figure 2B presents a bubble plot of the GO enrichment analysis results for the DEGs, featuring the top 10 significantly enriched pathways. The size of each bubble indicates the number of genes involved in the process, while the color reflects the negative logarithm of the corrected p-value—darker colors denote higher significance. GO enrichment analysis revealed that characteristic genes predominantly participate in processes such as regulating the cell cycle (negative regulation of the cell cycle), chromosome segregation (including chromosome segregation, chromosomal regions, and centromeres), and microtubule structure (microtubules, microtubule binding, motor activity). KEGG pathway analysis indicated that these genes are enriched in pathways related to cell cycle dysregulation (cell cycle, aminoacyl-tRNA biosynthesis), DNA repair defects (DNA replication), hormone signaling abnormalities (progesterone-mediated oocyte maturation), p53 signaling pathways, and metabolic processes (pyrimidine metabolism) (Figures 2C,D). These genes may influence cellular structure and chromosomal dynamics, findings consistent with previous studies on adenomyosis (Yang et al., 2025). This is related to abnormal proliferation of tissue cells and disordered cell cycle regulation in adenomyosis, which may reflect abnormal cell proliferation or regulation in the disease. Targeted intervention on these pathways may become a future therapeutic strategy.

### 3.2 Construction of weighted gene Co-expression networks and identification of key modules using WGCNA

**FIGURE 3:**
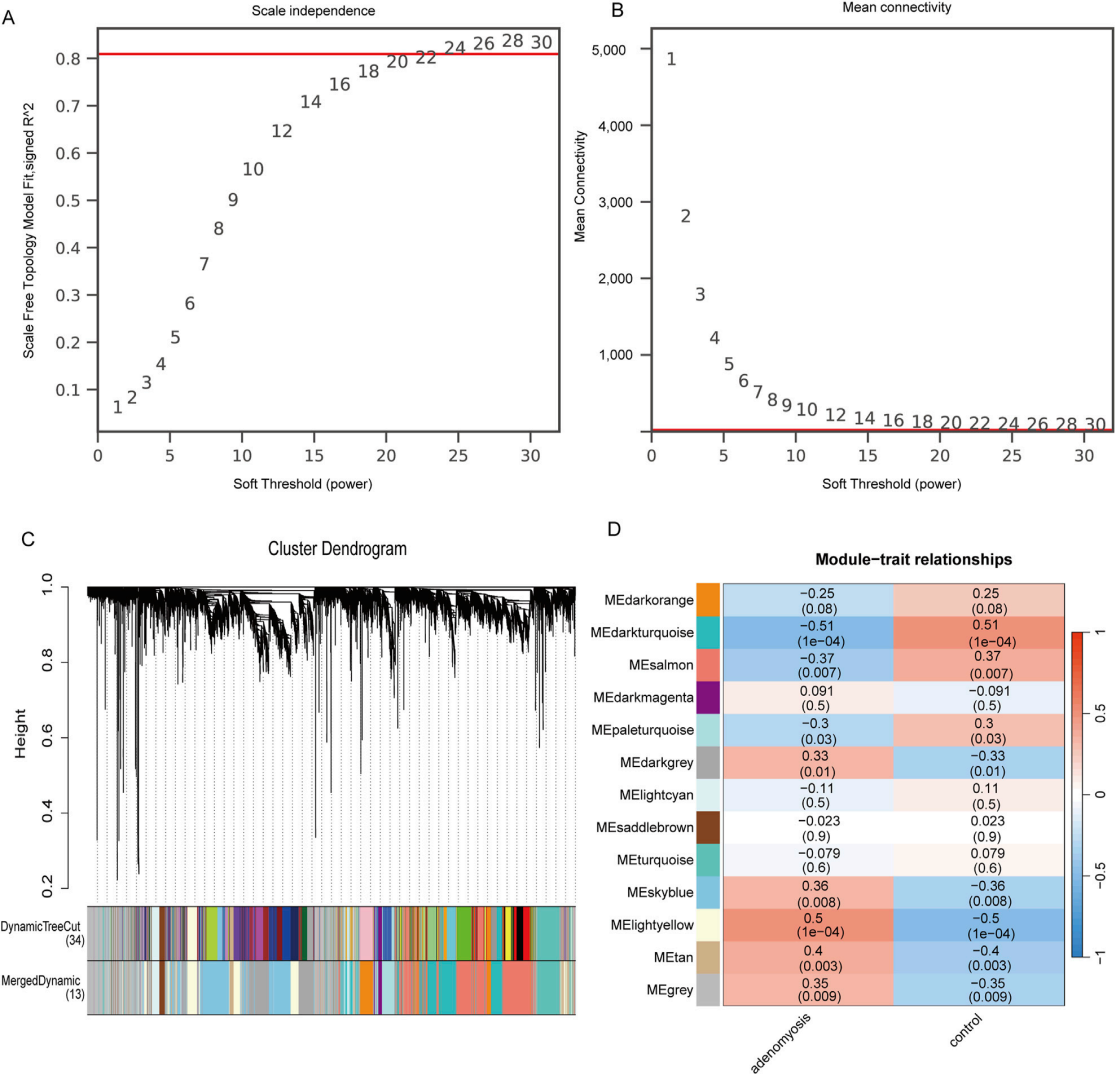
Construction of gene co-expression networks associated with adenomyosis through WGCNA. **(A)** Selection of soft threshold power. **(B)** Shows the change in average connectivity with different soft thresholds. (Soft threshold (power = 24) and scale-free topology fit index (R2 = 0.81)) **(C)**. Gene hierarchy tree-clustering diagram. The graph indicates different genes horizontally and the uncorrelatedness between genes vertically, the lower the branch, the less uncorrelated the genes within the branch, i.e., the stronger the correlation. **(D)** Heatmap showing the relations between the module and adenomyosis features. The values in the small cells of the graph represent the two-calculated correlation values cor coefficients between the eigenvalues of each trait and each module as well as the corresponding statistically significant p-values. Color corresponds to the size of the correlation; the darker the red, the more positive the correlation; the darker the Blue, the more negative the correlation.

To explore the co-expression network associated with adenomyosis, we constructed a weighted gene co-expression network using the WGCNA method based on the dataset GSE244236. Using the unscaled topology criterion (*R* ^2^ = 0.81), we determined the optimal soft threshold power as 24 while ensuring that the average connectivity remained close to zero (Figures 3A,B). The selected soft threshold was then applied to construct a co-expression matrix, with a minimum module size of 50 genes, a dynamic tree-cutting parameter set to 2, and a module merging threshold of 0.25. Different gene modules were assigned distinct color labels. Subsequently, gene hierarchical clustering trees were created through gene correlation analysis, identifying 13 differently colored modules, with the gray module representing gene sets that were deemed ineligible for classification (Figure 3C). Finally, considering correlation coefficients, p-values, and module GS/MM values, we identified the MElightyellow module as positively correlated with adenomyosis and regarded as the disease characteristic; the genes within this module were designated as key genes (Figure 3D). Adenomyosis showed a positive correlation with these key genes (*r* ^2^ = 0.50, p = 1E−04).

### 3.3 Enrichment analysis of crossover genes

**FIGURE 4:**
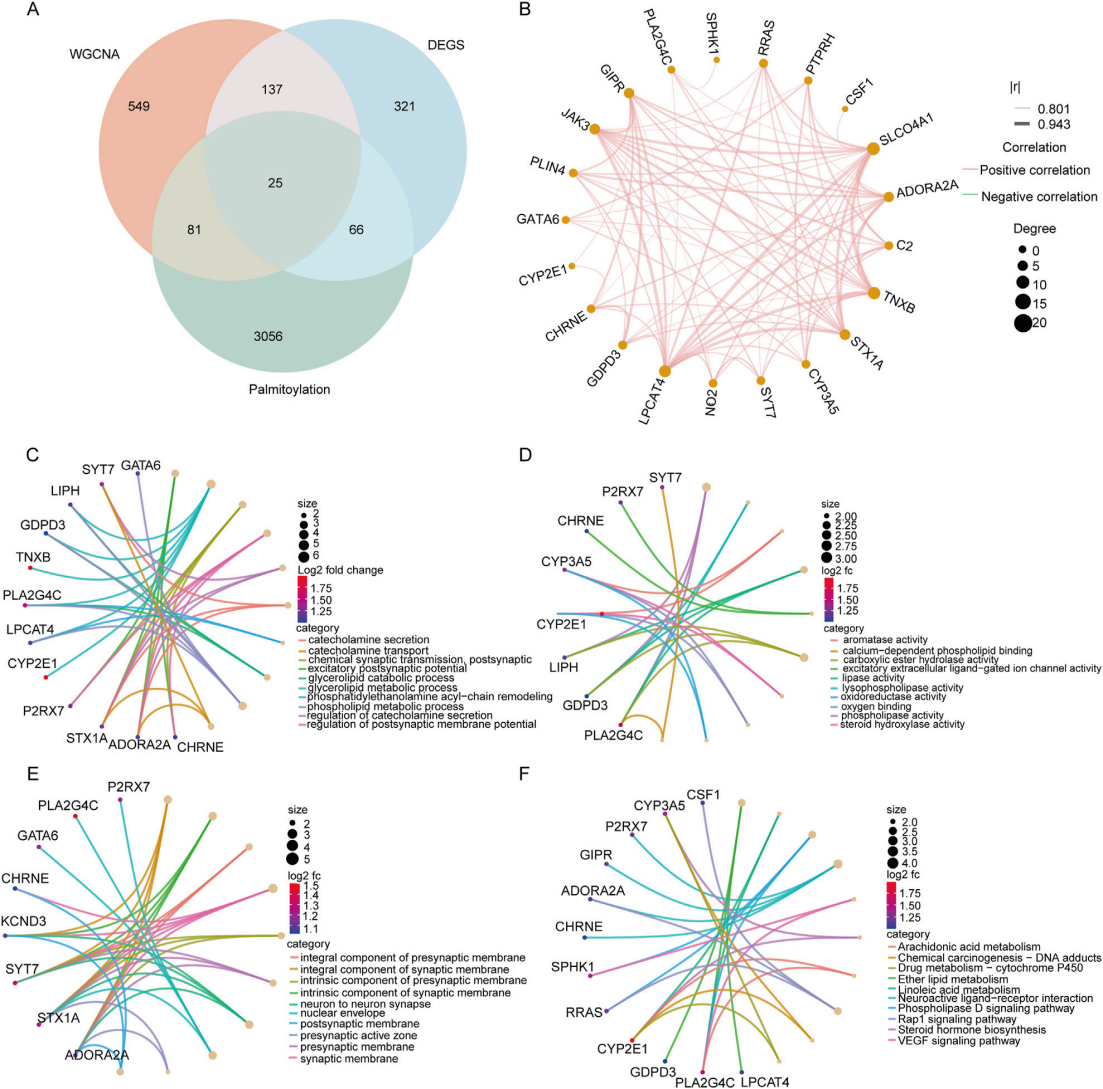
Pathway enrichment analysis of the intersecting genes selected. **(A)** Venn diagram of 25 genes screened using 549 DEGS, 3228 palmitoylated genes and MElightyellow modular genes of WGCNA. **(B)** Correlation analysis of 25 genes; **(C–E)**: GO functional annotation of signature genes. The top 10 BP, MF, and MF terms of DEGs. **(F)** Functional annotation of the Kegg signaling pathway of signature genes.

To investigate the regulatory role of palmitoylation in the pathogenesis of adenomyosis, we intersected DEGs, MElightyellow genes, and palmitoylation-related genes, resulting in a total of 25 characteristic genes (Figure 4A). Figure 4B depicts the correlation network of these 25 genes, which indicates that they are all positively correlated. Subsequently, we conducted functional enrichment analysis on these characteristic genes to explore their biological functions and potential pathways involved in palmitoylation during adenomyosis (Figures 4C–F). The GO enrichment analysis revealed that these genes are primarily involved in biological processes such as neurotransmitter secretion and synaptic transmission (including catecholamine secretion, chemical synaptic transmission, and postsynaptic processes), glycerolipid metabolism (covering glycerolipid catabolic and metabolic processes), and steroid metabolism (including aromatase activity). KEGG pathway analysis indicated that these genes are mainly enriched in pathways related to lipid metabolism and signaling (such as arachidonic acid metabolism), steroid hormone biosynthesis, drug metabolism (via cytochrome P450), neuroactive ligand-receptor interactions, and VEGF signaling pathways. Current research suggests that palmitoylation regulates the cell cycle through protein membrane localization and stability, thereby significantly influencing inflammatory responses and lipid metabolism (Qu et al., 2021). This supports the hypothesis that palmitoylation may contribute to the development of adenomyosis by modulating lipid metabolism, hormone synthesis, neural signaling, and angiogenesis processes.

### 3.4 Identification of diagnostic biomarkers

**FIGURE 5:**
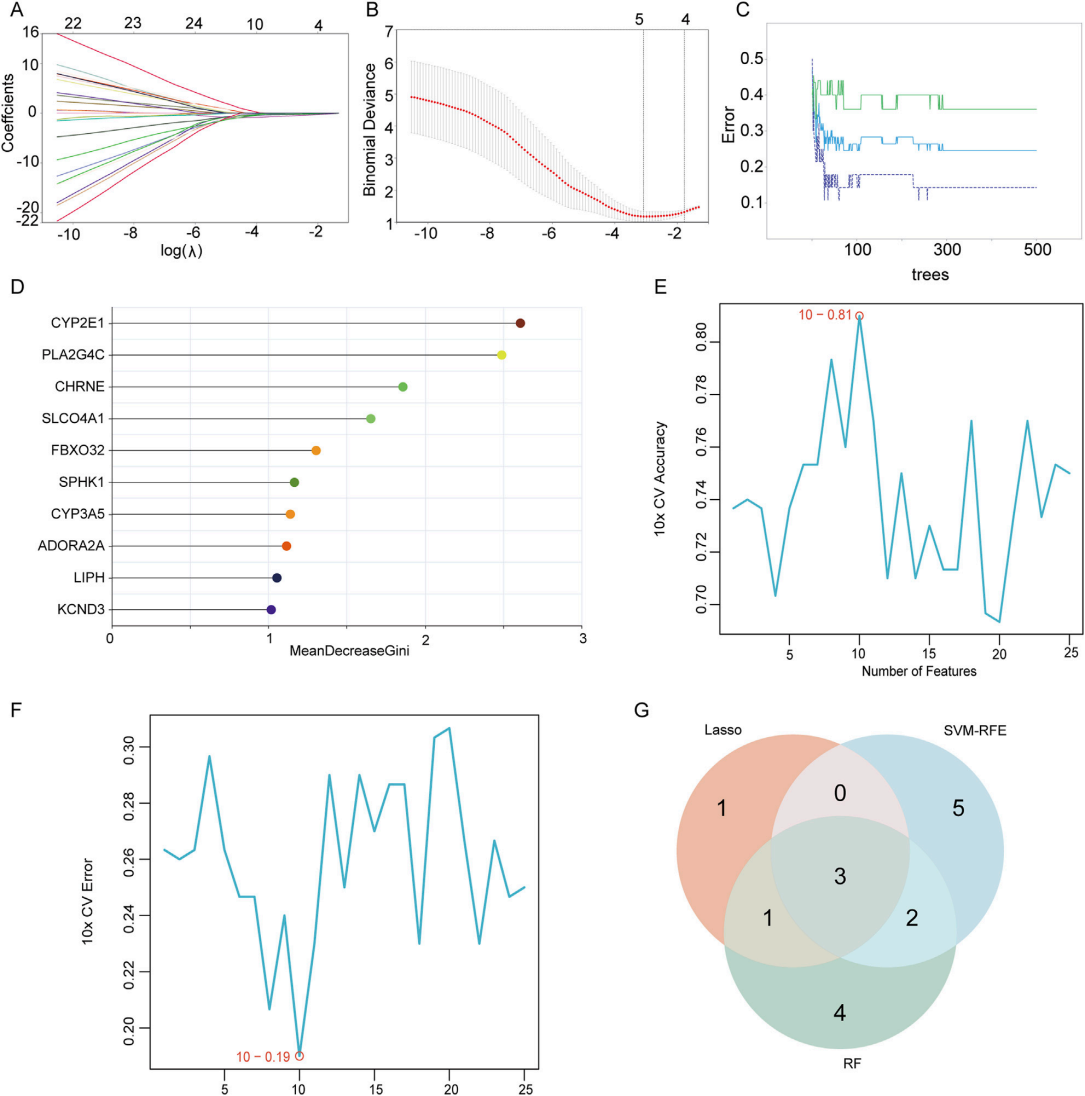
Candidate diagnostic biomarkers were identified by an integrated strategy. **(A,B)** Significant prognostic variables screened using LASSO regression. **(C,D)** Top 10 significant genes screened using RF. **(E,F)** Images screened using SVM-RFE. **(G)** Venn diagram of candidate diagnostic biomarkers screened using LASSO, RF and SVM-RFE.

To identify potential diagnostic biomarkers from these 25 key genes, we integrated three advanced machine learning algorithms for joint screening analysis: LASSO, RF, and SVM-RFE. Based on the value of λmin, we performed LASSO regression analysis and identified five hub genes as the most representative markers associated with adenomyosis development (Figures 5A,B). In the RF algorithm, we set ntree to 500 to stabilize the model errors, thereby selecting the top 10 most significant genes as final candidates (Figures 5C,D). The top 10 genes identified by SVM-RFE showed the highest significance, achieving an accuracy of 0.81 (Figure 5E) and a false positive rate of 0.19 (Figure 5F). Subsequent intersection analysis using Venn diagrams (Figure 5G) among the results of SVM-RFE, LASSO, and RF identified three diagnostic biomarkers: LIPH, CYP2E1, and CHRNE. These biomarkers offer novel molecular targets for early diagnosis and intervention in adenomyosis, demonstrating substantial clinical value.

### 3.5 Modeling of diagnostic biomarkers

**FIGURE 6:**
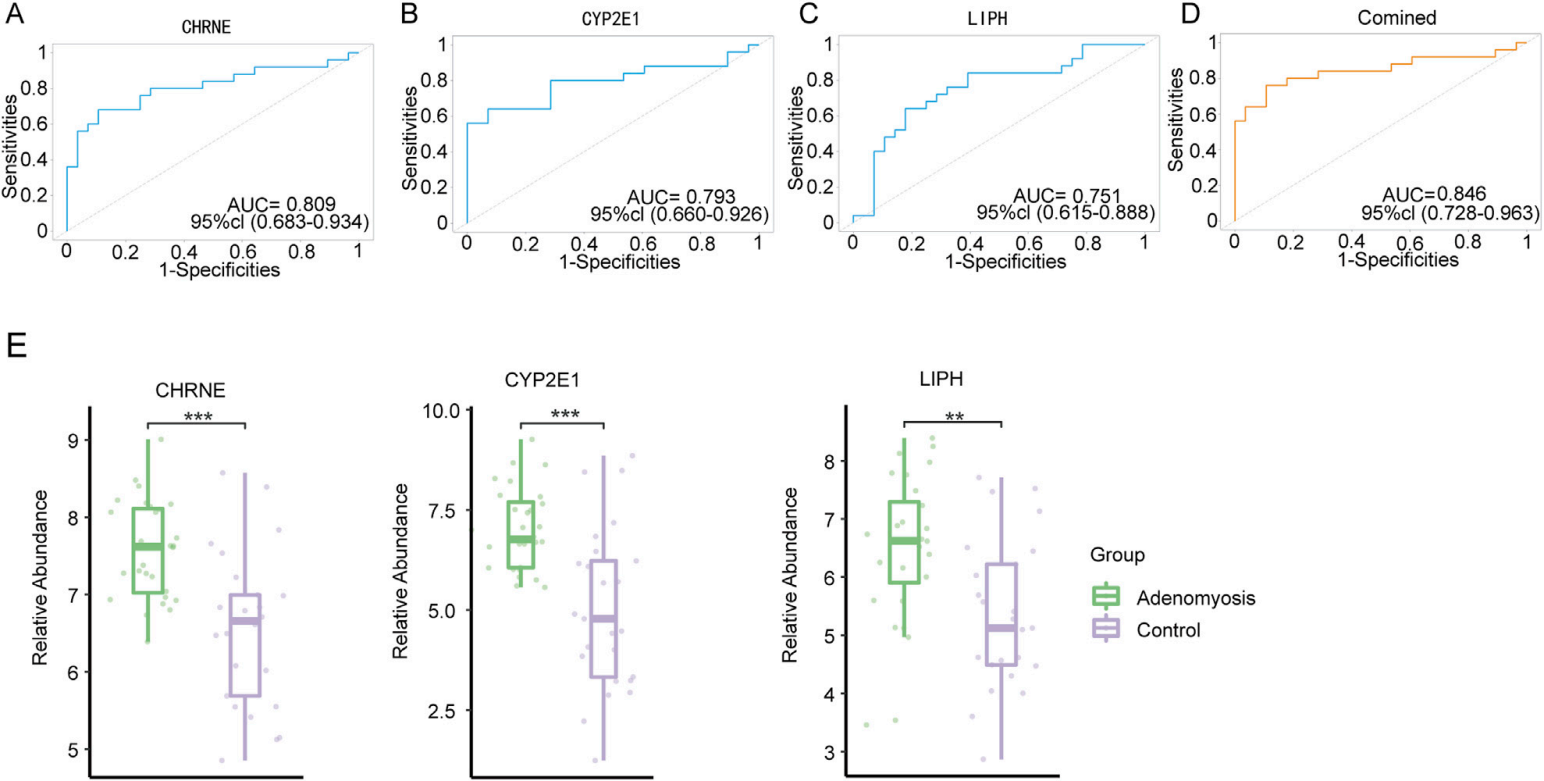
Construction of diagnostic models. **(A-C)** ROC curve analysis and AUC were calculated for CHRNE, CYP2E1, and LIPH in 53 samples. **(D)** ROC curves validating the diagnostic efficacy of the three co-diagnostic markers using logistic regression analysis. **(E)** Expression of CHRNE, CYP2E1, and LIPH in the GSE244236 dataset (*p < 0.05, **p < 0.01, ***p < 0.001).

To systematically evaluate the performance of the three genes as diagnostic biomarkers, we plotted ROC curves and used the area under the curve (AUC) as a key indicator of model prediction accuracy. Figures 6A–C display the ROC analysis results for these genes within the GSE244236 dataset. The results indicated that each gene exhibited good diagnostic value, with AUC values exceeding 0.7, suggesting high diagnostic accuracy when used individually as biomarkers. Furthermore, we evaluated the diagnostic efficacy of combining these biomarkers. The combined analysis showed a significant improvement, with an AUC of 0.846 (Figure 6D). These findings demonstrate that the diagnosis of adenomyosis can be achieved more reliably through the combined use of these biomarkers, supporting their clinical applicability.

To further assess the diagnostic potential of LIPH, CYP2E1, and CHRNE in GSE244236 using ROC analysis, we used an AUC >0.7 as the inclusion criterion to improve diagnostic performance. In ROC curve analyses (Figures 6A–C), the AUC values of these three genes indicated strong sensitivity and specificity in diagnosing adenomyosis. Box plot visualizations of gene expression patterns between adenomyosis and control groups further confirmed their high diagnostic potential (Figure 6E). Additionally, Figure 6D illustrates the combined diagnostic value of these three genes, further validating their effectiveness and reliability as biomarkers, especially when used synergistically.

### 3.6 Validation of diagnostic biomarkers

**FIGURE 7:**
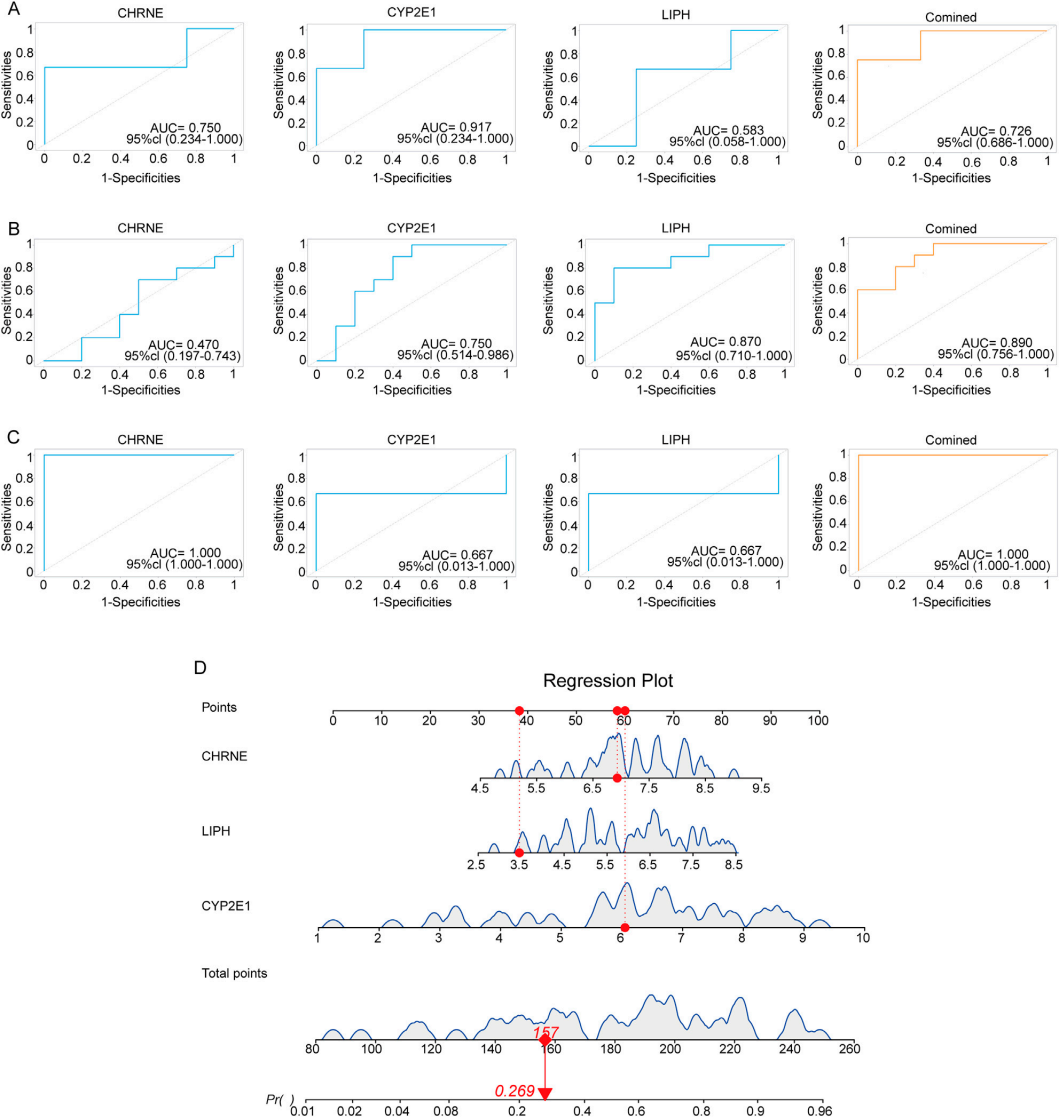
Validation of genetic diagnostic models using three public GEO datasets. **(A)** ROC plots of three diagnostic genes in the GSE190580 dataset using area under the curve (AUC) to assess model accuracy. **(B)** ROC plots of three diagnostic genes in the GSE185392 dataset using area under the curve (AUC) to assess model accuracy. **(C)** ROC plots of three diagnostic genes in the GSE157718 dataset, using area under the curve (AUC) to assess model accuracy. **(D)** Nomogram of the diagnostic model using the three characterized genes.

To further validate the reliability and clinical application value of the three selected diagnostic genes, we conducted AUC curve analysis on three GEO datasets (GSE185392, GSE190580, GSE157718). In the GSE190580 dataset (Figure 7A), the AUC values for these three genes were 0.58, 0.91, and 0.75, while the combined ROC area under the curve achieved 0.726, further confirming the reliability of the results. In the GSE185392 dataset (Figure 7B), the AUC values for LIPH, CYP2E1, and CHRNE were 0.87, 0.75, and 0.47. When performing a combined analysis of these three genes, the AUC value reached 0.89, indicating potential enhanced performance through integrated analysis. Similar outcomes were observed in the GSE157718 dataset (Figure 7C), where the AUC values for these three genes were 0.667, 0.667 and 1.0 respectively. These analyses support the clinical diagnostic potential of the selected genes, particularly the combined analysis results, which could serve as candidate molecular markers for disease-assisted diagnosis. While this study has validated the diagnostic potential of candidate genes across multiple independent datasets, some genes demonstrated suboptimal performance in specific cohorts, suggesting their diagnostic efficacy may be influenced by cohort-specific factors. Future research should further investigate the robustness and clinical applicability of these genes through larger-scale, multicenter, prospective clinical trials.

To enhance clinical applicability, we visualized the nomograms using the “regplot” package. This plot allows the total score of the three genes to be converted into a probability value (Pr) that predicts the risk of disease. For example, Figure 7D shows a Pr of 0.269, indicating a 26.9% probability of adenomyosis based on these gene expressions. These analyses not only verified the reliability of the three genes as diagnostic biomarkers but also demonstrated their potential clinical value through column line diagrams, providing a new molecular basis for early diagnosis and treatment of adenomyosis.

### 3.7 ssGSEA immune cell infiltration analysis

**FIGURE 8:**
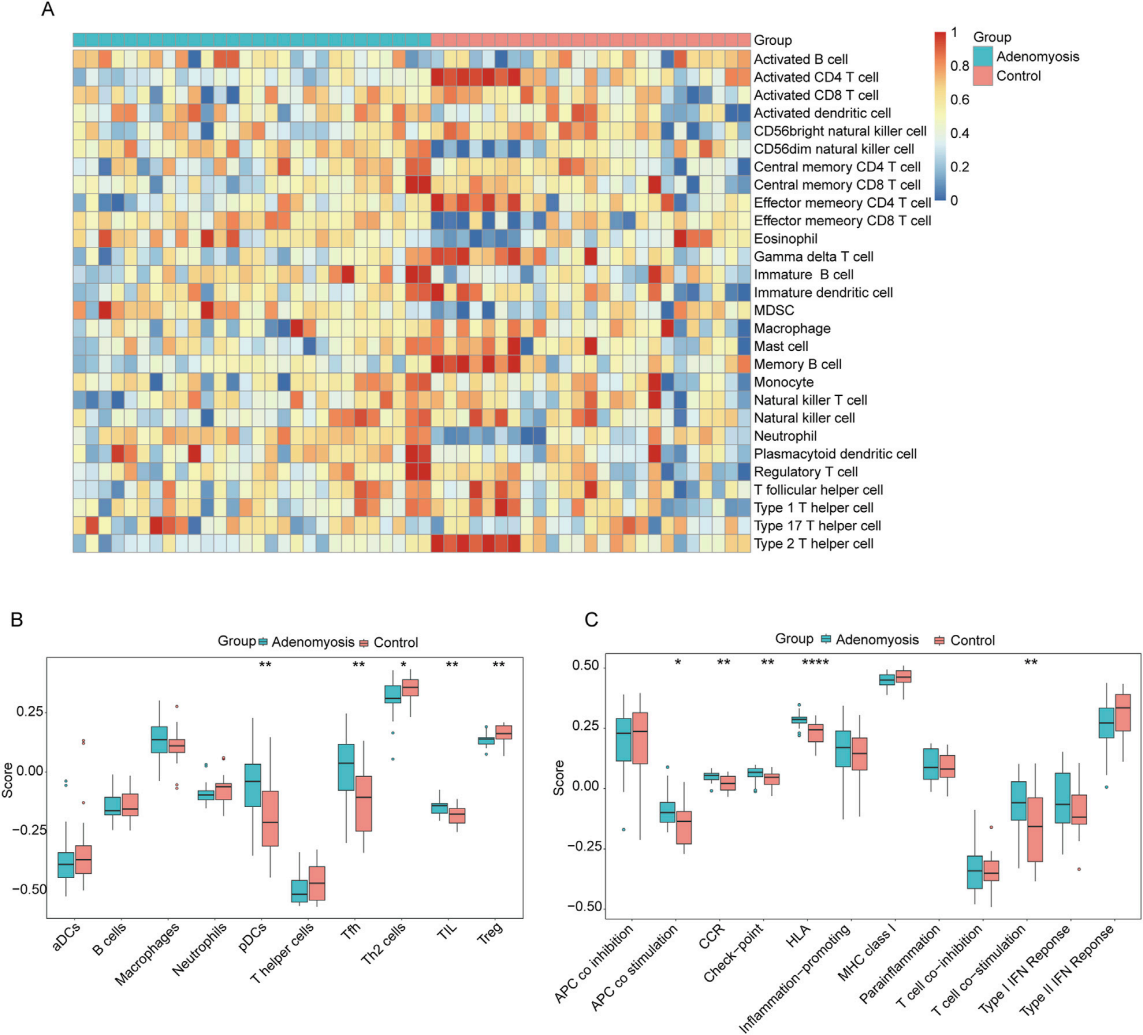
Immune cell infiltration analysis using ssGSEA. **(A)** Heatmap of the distribution of 28 immune cells in the adenomyosis group and control group. **(B)** Box line plot of the distribution of 16 immune cells in the adenomyosis group and control group. **(C)** Box line plots of the distribution of 13 immune features in the adenomyosis group and the control group (*p < 0.05, **p < 0.01, ***p < 0.001, ****p < 0.0001).

We observed that, in samples from patients with adenomyosis, the infiltration rates of CD56 dim natural killer cells, effector memory CD8 T cells, eosinophils, immature B cells, myeloid-derived suppressor cells (MDSCs), neutrophils, and plasmacytoid dendritic cells were significantly higher, suggesting that these immune cells play a key role in the progression of adenomyosis (Figure 8A).

To further analyze the relationship between adenomyosis and immune infiltration in depth, we performed a comparative study of 16 immune cell infiltration types and 13 immune characteristics (Figures 8B, C). The results indicated that, compared with the control group, the proportions of plasmacytoid dendritic cells (pDCs), follicular helper T cells (Tfh), and tumor-infiltrating lymphocytes (TILs) were relatively high in the adenomyosis lesion tissues, while the proportions of regulatory T cells (Tregs) and Th2 cells were relatively low. Among the 13 immune characteristics, antigen-presenting cell (APC) co-stimulation, chemokine receptor (CCR), immune checkpoint molecules, human leukocyte antigen (HLA), Th2 cells, and Treg levels were more prevalent in the adenomyosis group. These findings indicate that there are significant differences in immune infiltration between the adenomyosis lesion group and the control group, with overall immune infiltration levels being lower in the adenomyosis tissues compared to normal tissues.

### 3.8 Correlation analysis between diagnostic markers and infiltration-associated immune cells

To thoroughly investigate potential associations between immune cell infiltration and diagnostic biomarkers in adenomyosis, we systematically analyzed the correlations between 28 immune cell types and three diagnostic markers (LIPH, CYP2E1, and CHRNE). Additionally, to explore whether palmitoylation is involved in the micro-regulation of immune infiltration in adenomyosis, we examined the association between the palmitoylation hub genes and immune cell infiltration based on the GSE244236 dataset.

**FIGURE 9:**
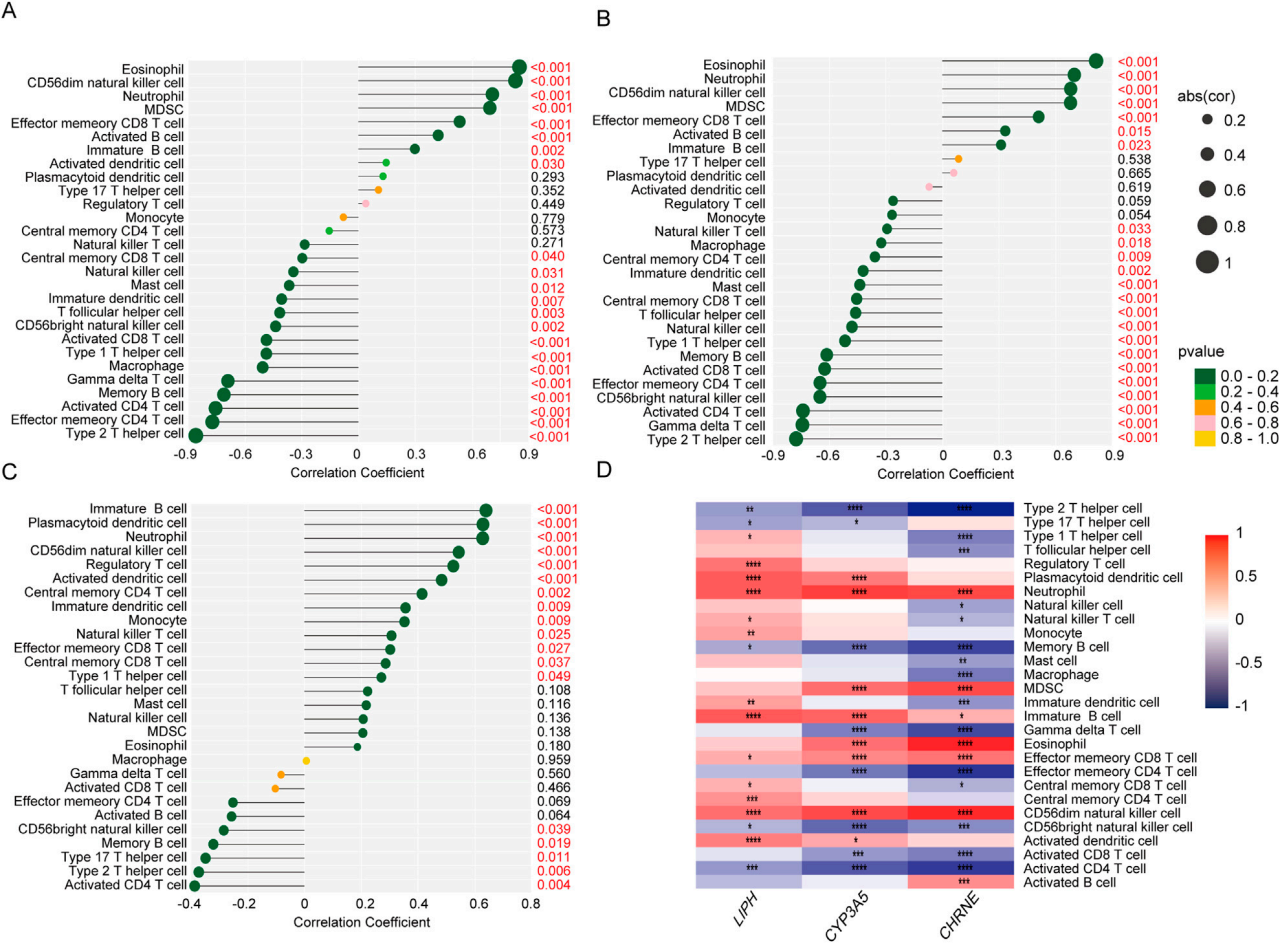
Correlations between three diagnostic biomarkers and infiltrating immune cells were analyzed. **(A)** Correlation between LIPH infiltrating immune cells. **(B)** Correlation between CYP2E1 infiltrating immune cells. **(C)** Correlation between CHRNE infiltrating immune cells. **(D)** Analysis of the correlation between core genes and immune functional pathways.

Figures 9A–C display the correlation between 28 immune cell types and the three genes using lollipop plots. To present a clearer picture of the relationship between these diagnostic markers and immune infiltration, we employed an immune infiltration heatmap (Figure 9D). The heatmap showed positive correlations between all three genes and effector neutrophils, immature B cells, effector memory CD8 T cells, and CD56 dim natural killer cells. Conversely, there were negative correlations between the three genes and type 2 helper T cells (Th2), memory B cells, CD56 bright natural killer cells, and activated CD4 T cells.

The findings of this study demonstrate a strong correlation between these three biomarkers and impaired immune cell infiltration in adenomyosis, suggesting that these genes may play a significant role in the disease’s immunopathological mechanisms. This provides crucial insights for further research and potential clinical applications. However, the causal relationship between palmitoylation genes and the immune microenvironment remains unclear. Future studies could employ causal inference models to explore their interplay, thereby clarifying their specific roles in adenomyosis pathogenesis. Additionally, validating the candidate genes’ specific expression patterns in epithelial, stromal, or immune cells using single-cell RNA sequencing datasets will help deepen our understanding of their regulatory functions within the immune microenvironment.

## 4 Discussion

Adenomyosis is a common gynecologic disorder affecting women of reproductive age, characterized by the invasion of the endometrium into the myometrium. This condition often manifests as chronic pelvic pain, abnormal uterine bleeding, and infertility. Currently, adenomyosis is diagnosed through vaginal ultrasonography (Krentel et al., 2023), magnetic resonance imaging, and biomarker testing. However, the pathogenesis of the disease remains incompletely understood. Therefore, it is essential to identify high-quality diagnostic markers for adenomyosis to enhance both diagnosis and treatment. Additionally, patients with adenomyosis are at risk of disease progression and may develop malignant conditions such as endometrial cancer, ovarian cancer, uterine sarcoma, and clear cell carcinoma (Moraru et al., 2023; Tabuchi et al., 2025). Therefore, early diagnosis is crucial for ensuring therapeutic efficacy and improving quality of life. The aim of this study was to investigate the expression of palmitoylation-related genes in adenomyosis and to identify potential diagnostic biomarkers.

Palmitoylation is a important protein modification process that plays a crucial role in variety diseases, including cancer, metabolic diseases, and neurodegenerative diseases, by regulating the cellular localization of proteins, signaling pathways, and protein interactions. However, the research on palmitoylation-related genes and their mechanisms in gynecological diseases is limited. In the context of adenomyosis, estrogen may promote the depalmitoylation of Scribble by up-regulating the expression of APT1 and APT2, resulting in its translocation from the cell membrane to the cytoplasm. Consequently, undertaking a comprehensive study of adenomyosis diagnosis and treatment from the perspective of palmitoylation genes holds significant scientific importance.

In this study, we developed a genetic diagnostic model based on the GEO public datasets, integrating comprehensive bioinformatics analysis and various machine learning algorithms. First, we used the GSE244236 dataset as a training set to compare adenomyosis samples with control samples, identifying 549 differentially expressed genes (DEGs), of which 302 were upregulated and 247 downregulated. GO analysis indicated that these DEGs are primarily involved in regulating the cell cycle, chromosome segregation, and microtubule structure. These functions are related to abnormal tissue cell proliferation and disrupted cell cycle regulation in adenomyosis, reflecting the molecular mechanisms underlying abnormal cell growth in the disease. Furthermore, we utilized WGCNA to construct gene co-expression networks, clustering genes with similar expression patterns and identifying modules closely associated with clinical traits. Through integrative analysis, we identified MElightyellow gene modules significantly correlated with adenomyosis. Incorporating 3,228 palmitoylated genes, we found intersections between these and the DEGs, resulting in 25 key genes that served as the basis for further analysis. In the subsequent screening phase, we combined three machine learning algorithms—LASSO, RF, and SVM-RFE—to identify three potential diagnostic biomarkers: LIPH, CYP2E1, and CHRNE. Validation across three independent datasets using ROC analysis demonstrated the high diagnostic accuracy of these biomarkers. The combined use of the three markers further improved diagnostic performance, supporting our original hypothesis. Additionally, we constructed a diagnostic model visualized through nomogram to evaluate its predictive accuracy. We also explored immune cell infiltration characteristics in adenomyosis and examined their correlation with the three biomarkers, providing insights into the immunopathological mechanisms involved.

Through bioinformatics analysis, this study identified three key genes closely associated with adenomyosis: LIPH, CYP2E1, and CHRNE. LIPH, which encodes an endothelial lipase, regulates lipid metabolism and the lysophosphatidic acid (LPA) signaling pathway, and is involved in processes related to tumors, inflammatory responses, and hereditary hair disorders (Han et al., 2023; Jiang et al., 2025). Although no direct studies have yet confirmed the role of LIPH in adenomyosis, lipid metabolism disorder is considered a key feature of the disease. Elevated local estrogen levels may establish a positive feedback loop with lipid metabolism, promoting abnormal proliferation and invasion of endometrial cells (Harada et al., 2016). We hypothesize that LIPH may contribute to adenomyosis development by regulating lipid metabolism pathways. CYP2E1, a member of the cytochrome P450 family involved in lipid metabolism, plays a critical role in oxidative stress and inflammatory responses. Current research mainly focuses on its associations with liver diseases, inflammatory disorders, cancer, and neurological conditions (Han et al., 2024; Jung et al., 2024; Ye et al., 2021). In adenomyosis, the high expression of P450 aromatase facilitates local estrogen synthesis, which activates inflammatory and oxidative stress pathways, thereby promoting disease progression. The C-1054T polymorphism (rs2031920) in the CYP2E1 promoter region has shown a significant association with the risk of polycystic ovary syndrome (PCOS) in Chinese Han women, suggesting its important role in metabolic and inflammatory disorders (Pu et al., 2023). It is hypothesized that CYP2E1 may participate in the pathogenesis of adenomyosis by modulating oxidative stress and inflammatory responses. CHRNE, which encodes the epsilon subunit of cholinergic receptors, plays a role in neuromuscular signal transduction. Emerging evidence indicates that neuropeptide signaling and neurotransmitter receptor pathways may contribute to adenomyosis development. Acetylcholine, acting through its α7-nicotinic acetylcholine receptor (α7nAChR), can exert anti-inflammatory effects and potentially inhibit disease progression (Xu et al., 2021). Studies have also shown that α7-nAChR agonists can slow the progression of endometriosis by suppressing inflammatory responses (Hao et al., 2022). Therefore, it is hypothesized that CHRNE may influence disease occurrence and progression through immune regulation mechanisms, modulation of uterine smooth muscle excitability, and alterations in the local immune microenvironment. Future mechanistic studies are needed to verify these hypotheses and explore the potential of CHRNE as a therapeutic target.

The potential roles of these genes in adenomyosis represent novel findings of our study; however, further data and experimental validation are necessary. To investigate their possible functions, we analyzed the palmitoylation profiles of LIPH, CYP2E1, and CHRNE using the SwissPalm database. Results indicate that LIPH has a predicted palmitoylation site at Cys-13, but this has not yet been validated experimentally. For CYP2E1, there is no mass spectrometry evidence of palmitoylation in humans, although data from mouse models suggest potential modification. Regarding CHRNE, palmitoylation has only been reported at neuromuscular junctions, with no validation in other tissues. Collectively, all three genes possess potential palmitoylation sites, but direct experimental evidence remains lacking. Future studies should incorporate functional experiments to clarify their regulatory roles in adenomyosis. Additionally, these gene expression changes may be influenced by the immune microenvironment of adenomyosis, which warrants further exploration through genetic approaches such as Mendelian randomization and single-cell multi-omics techniques to elucidate this bidirectional interaction network.

Despite the findings presented in this study, several limitations must be acknowledged. First, the dataset used has a relatively small sample size and includes data collected from different platforms, emphasizing the need for validation in larger, independent cohorts. Second, additional *in vivo* and *in vitro* experiments are essential to confirm the roles of these biomarkers in the pathogenesis of adenomyosis and their association with immune infiltration. Finally, the clinical utility and specificity of these biomarkers require further validation through prospective clinical studies.

In summary, this study identified three potential diagnostic biomarkers by integrating bioinformatics analyses with multiple machine learning algorithms, providing preliminary insights into their biological functions and diagnostic potential in adenomyosis. These findings lay the groundwork for future research and hold promise for improving the diagnosis and treatment strategies for adenomyosis.

## Data Availability

The datasets presented in this study can be found in online repositories. The names of the repository/repositories and accession number(s) can be found below: GSE244236, GSE190580, GSE185392 and GSE157718.
