# Correction: Analysis and validation of novel biomarkers related to palmitoylation in adenomyosis

**DOI:** 10.3389/fgene.2025.1683914

**Published:** 2025-08-21

**Authors:** Hongyu Zhang, Yufeng Li, Huijuan Cao, Yiling Zhao, Hongwen Zhu, Tiansheng Qin

**Affiliations:** ^1^ Department of Gynecology, The Second Hospital & Clinical Medical School, Lanzhou University, Lanzhou, China; ^2^ Cuiying Biomedical Research Center, The Second Hospital & Clinical Medical School, Lanzhou University, Lanzhou, China

**Keywords:** palmitoylation, adenomyosis, machine learning, diagnostic model, gene expression

In the published article, there were inconsistencies in the count of patients with adenomyosis and normal controls. The study includes a total of 53 samples: 28 adenomyosis cases and 25 normal controls.

A correction has been made to section **2 Materials and methods**, *2.1 Data set collection and processing*:

“We obtained the gene expression profiles of adenomyosis from the GEO database, including four microarray datasets: GSE244236, GSE190580, GSE185392, and GSE157718. The GSE244236 dataset contains the expression profiles of 28 patients with adenomyosis and 25 normal controls. The remaining three datasets contain endometrial expression profiles of different numbers of adenomyosis patients and controls, respectively. We set GSE244236 as the training set and the remaining three datasets as the validation set. In addition, we downloaded 3228 palmitoylation-related genes through the GeneCards website (**Zeng et al., 2024**).”

A correction has been made to section **2 Materials and methods**, *2.2 Identification and functional enrichment analysis of differentially expressed genes*:

“After performing gene re-annotation on the dataset probes using platform files, all data underwent logarithmic transformation and normalization through the normalizeBetweenArrays function. The Limma package was then employed to identify DEGs between 28 women with adenomyosis and 25 healthy controls, using the threshold for significant differences at adjusted p-values <0.05 and |log2(FC)| ≥ 1, which resulted in the identification of 549 DEGs (**Figure 2A**). To provide a visual overview of these DEGs, subsequent visualization involved volcano plot analysis (bioinformatics.com.cn). In order to understand the functional implications of these DEGs, we conducted functional enrichment analyses. Through Gene Ontology (GO) analysis, we examined biological processes (BP), cellular components (CC), and molecular functions (MF) associated with the DEGs. Additionally, Kyoto Encyclopedia of Genes and Genomes (KEGG) enrichment analysis was applied to explore pathway enrichment (**Figures 2B–D**).”

A correction has been made to section **3 Results**, *3.1 Screening and visualization of differentially expressed genes*, Paragraph 2:

“The dataset GSE244236 was sourced from the Gene Expression Omnibus (GEO) database. Using probe annotation information, we converted integrated IDs in the gene expression matrix into gene symbols. The expression matrices were then normalized through log2 transformation and processed using the normalizeBetweenArrays function. Differential analysis was performed with the limma package to compare 28 adenomyosis patients with 25 normal controls, identifying 549 DEGs with adjusted p-values <0.05 and |log2(FC)| ≥ 1, visualized through volcano plots (**Figure 2A**). Among these DEGs, 302 genes showed upregulation (indicated by red dots), while 247 showed downregulation (indicated by blue dots). To elucidate their functional significance, we conducted Gene Ontology (GO) and Kyoto Encyclopedia of Genes and Genomes (KEGG) pathway enrichment analyses using platform (http://www.bioinformatics.com.cn), with a significance threshold of P < 0.05.”

In the published article, there were also inconsistencies in the number of potential diagnostic genes. The correct number is three.

A correction has been made to the Results section of the **Abstract**:

“Results: A total of 549 differentially expressed genes were identified in the analysis. Through WGCNA analysis, we obtained 25 palmitoylation-related intersecting genes. Using LASSO, RF and SVM-RFE algorithms, three potential diagnostic genes were finally screened: LIPH, CYP2E1 and CHRNE.”

A correction has been made to section **3 Results**, *3.6 Validation of diagnostic biomarkers*, Paragraph 1:

“To further validate the reliability and clinical application value of the three selected diagnostic genes, we conducted AUC curve analysis on three GEO datasets (GSE185392, GSE190580, GSE157718). In the GSE190580 dataset (**Figure 7A**), the AUC values for these three genes were 0.58, 0.91, and 0.75, while the combined ROC area under the curve achieved 0.726, further confirming the reliability of the results. In the GSE185392 dataset (**Figure 7B**), the AUC values for LIPH, CYP2E1, and CHRNE were 0.87, 0.75, and 0.47. When performing a combined analysis of these three genes, the AUC value reached 0.89, indicating potential enhanced performance through integrated analysis. Similar outcomes were observed in the GSE157718 dataset (**Figure 7C**), where the AUC values for these three genes were 0.667, 0.667 and 1.0 respectively. These analyses support the clinical diagnostic potential of the selected genes, particularly the combined analysis results, which could serve as candidate molecular markers for disease-assisted diagnosis. While this study has validated the diagnostic potential of candidate genes across multiple independent datasets, some genes demonstrated suboptimal performance in specific cohorts, suggesting their diagnostic efficacy may be influenced by cohort-specific factors. Future research should further investigate the robustness and clinical applicability of these genes through larger-scale, multicenter, prospective clinical trials.”

Finally, the textual descriptions of **Figures 5C–F** in Section 3.4 appeared in the wrong order in the published article.

A correction has been made to section **3 Results**, *3.4 Identification of diagnostic biomarkers*:

“To identify potential diagnostic biomarkers from these 25 key genes, we integrated three advanced machine learning algorithms for joint screening analysis: LASSO, RF, and SVM-RFE. Based on the value of λmin, we performed LASSO regression analysis and identified five hub genes as the most representative markers associated with adenomyosis development (**Figures 5A,B**). In the RF algorithm, we set ntree to 500 to stabilize the model errors, thereby selecting the top 10 most significant genes as final candidates (**Figures 5C,D**). The top 10 genes identified by SVM-RFE showed the highest significance, achieving an accuracy of 0.81 (**Figure 5E**) and a false positive rate of 0.19 (**Figure 5F**). Subsequent intersection analysis using Venn diagrams (**Figure 5G**) among the results of SVM-RFE, LASSO, and RF identified three diagnostic biomarkers: LIPH, CYP2E1, and CHRNE. These biomarkers offer novel molecular targets for early diagnosis and intervention in adenomyosis, demonstrating substantial clinical value.”

The original article has been updated.

